# Sixth Annual Report of the Registrar-General of Births, Deaths and Marriages in England

**Published:** 1845-07

**Authors:** 


I. SIXTH ANNUAL REPORT OF THE REGISTRAR-GENERAL OF BIRTHS, DEATHS, AND MARRIAGES IN ENGLAND. Folio, pp. 358. London, 1844.

II. REPORT ON THE SANATORY CONDITION OF NOTTINGHAM, COVENTRY, LEICESTER, DERBY, NORWICH AND PORTSMOUTH. By *James Ranald Martin*, Esq. Folio, pp. 58. London, 1844. (Health of Town's Commission.)

IN the number of this Journal for last July, our readers will find an abstract of the fifth Statistical Report drawn up by the Registrar-General. Although the present one does not contain so much medical information as most of its predecessors—the contributions of Mr. Farr being interesting rather to the actuary than to the physician—we trust that the data we shall adduce will be found useful to the profession generally, as bearing upon the important subject of Vital Statistics.

The number of Marriages, Births, and Deaths, registered in 1842, and in the three preceding years, were—

| | 1839 | 1840 | 1841 | 1842 |
|---|---|---|---|---|
| Marriages .. .. .. .. .. .. | 123,166 | 122,665 | 122,496 | 118,825 |
| Births .. .. .. .. .. .. .. .. | 492,574 | 502,303 | 512,158 | 517,739 |
| Deaths .. .. .. .. .. .. .. .. | 338,979 | 359,634 | 343,847 | 349,519 |
| Excess of Births over Deaths .. | 153,595 | 142,669 | 168,311 | 168,220 |

On the 1st of July 1841, the population of England was estimated to be 15,927,867—7,783,781 males, and 8,144,086 females. "The mean annual rate of increase in the 10 years (1831-1841) was 1·334 per cent.; and as all the births are not registered, and no correct account is kept of the numbers who come into or go out of the country from year to year, we have no other means of estimating the population living in each year, than to assume that the numbers increase at a uniform rate in geometrical progression. The estimate is of course not exactly correct; but the approximation to the true numbers is the nearest that can be made, and the addition or substraction of a few thousands will not materially affect the quotients of the rate of marriage, birth, or mortality, where the divisor is nearly 16,000,000."

From the following extract, it will be observed that the proportion of the Marriages, Births, and Deaths to the entire population, varied somewhat during these different years.

"To a mean population of 100, the average proportion of marriages was ·770, of births 3·200, and of deaths 2·209: or 1 person in 130 was married, 1 in 45 died, and 1 child was born to 31 persons living. These are the mean proportions of 4 years for marriages and births, of 5 years for deaths. The marriages decreased from 1 in 126 to 1 in 136. The births slightly increased up to the year 1841. The mortality was highest (2·290) in 1840, lowest (2·160) in 1841; but neither the rate of birth nor death differed sensibly in the two years 1841 and 1842."

It should always be remembered that the rate of mortality is sensibly higher in the male than it is in the female sex: in the former it is as 1 to 44, and in the latter as 1 to 47—or, more accurately, about 2,294 and 2,124 per cent.

The marriages, registered in England in 1842, were 3671 fewer than in 1841, and 4341 fewer than in 1839. The Registrar-General attributes the decrease of marriages in 1842, in part at least, to the depressed state of trade in that year. "As compared with 1841, the number of marriages 'not according to the rites of the Established Church,' increased 653, showing that more dissenters have availed themselves of the rights conceded to them by the Marriage Act of 1836. The marriages among Jews were 163: the marriages (113) of Jews the preceding year had been considerably below the average of 144 annually—a number of marriages which implies the existence of 18,700 Jews in England." Upon an average of the four years 1839-42, there has been one marriage annually to every 130 persons living—64 males and 66 females.

The following table exhibits the proportion of marriages to the population, in some of the great European States, as well as in England.

| | Years. | Population. | Marriages. | | | |
|---|---|---|---|---|---|---|
| | | Numbers | 1839 | 1840 | 1841 | 1842 |
| England* .. .. | 1841 | 15,927,867 | 123,166 | 122,665 | 122,496 | 118,825 |
| France . .. .. | 1841 | 34,213,920 | 266,890 | 281,998 | 283,902 | 280,412 |
| Austria (the German and Italian Provinces)† | 1840 | 21,571,594 | 168,872 | 169,322 | 184,122 | 176,999 |
| Prussia .. .. | 1840 | 14,928,501 | 128,676 | 132,281 | 138,168 | .. |
| Russia (part of) .. | 1842 | 49,525,420 | .. | .. | .. | 501,850 |
| | | 136,167,302 | | | | |

* Exclusive of Scotland and Ireland.

† The marriages in Hungary, Transylvania, and the Military Frontier, were estimated at 115,767 in 1839, which would make the marriages in the Austrian empire 234,639; the population in 1840 was about 36,950,401.

It would seem from this table (if we may depend upon its accuracy) that the relative proportion of marriages had been on the whole decreasing in England, and increasing in the continental states, from the year 1839 to 1842.

The subject of Marriage naturally leads us to that of

"*Births.*—2,024,774 births and 1,391,979 deaths were registered in 1839-42; so that the excess of births registered in four years amounted to 632,795. The mean annual number of births was 506,194, of deaths 347,995; and the annual addition to the population registered was on an average 158,199. The number of births registered in 1842 was 517,739, of deaths 349,519; and the excess of births over deaths was 168,220. At the rate of increase which prevailed in 1831-1841, the population would be 16,033,752 on 1st January, 1842, and 16,247,641 on 1st January, 1843; the increase would consequently be 213,889 in the year 1842."

The births registered in England are (it has been calculated), in proportion to the population, one-seventh part more numerous than in France, and one-seventh part less so than in Prussia. It is to be borne in mind that the registration of births was, a few years ago, and indeed still is, much less complete in this country than in most continental states; where indeed it is made compulsory by law.

According to the most accurate calculations, the average number of children to each married couple in England is very nearly *five*. Of this number about *three* survive to the age of marriage, to replace the two parents and those who have no offspring; the surplus either swelling the number of the general population, or emigrating to foreign lands.

*Number of Illegitimate Children.*—The first attempt to determine this statistical point in England was made by Mr. Rickman, at the Census of 1831. The total number then returned was 20,000; of whom 10,147 were males, and 9,892 females. But this estimate is certainly very much below the mark. "The number," says the Registrar-General, "of illegitimate children registered in 1842 amounted to 34,796; which is 14,757 —or 74 per cent.—more than the numbers in Mr. Rickman's Returns of 1830. The population increased only 17 per cent. in the 12 years. I am disposed to consider Mr. Rickman's Returns as deficient to a much greater extent than they were supposed to be at the period of their publication: but with a correction for the increase of population, the numbers in the Abstract for 1842 would only have exceeded those in Mr. Rickman's Returns for 1830 by 11,300 instead of 14,757. This difference may, perhaps, among other causes, be ascribed to an actual increase in the proportion of illegitimate children during the operation of that important change in the Poor Law, which threw the charge of maintaining their illegitimate offspring upon the mothers. To whatever cause the increase may be ascribed, the relative numbers of legitimate and illegitimate births and baptisms returned in 1830 and 1842, show in the latter year a relative as well as an absolute excess of illegitimate children."

*Relative Number of Male and Female Births.*—The number of boys born is, in all countries, greater than the number of girls. It has, moreover, been generally observed that the excess of males is rather greater among illegitimate than among legitimate children.

The annexed table shews the proportion of legitimate and illegitimate children born in several of the states of Europe, according to the latest returns which the Registrar-General has received. He very properly cautions the reader against hastily drawing from these numerical data any conclusions as to the comparative mortality, or otherwise, of the different countries. A passing allusion is made to "the peculiarity in the manners of the people, to which the paucity of children born out of wedlock in Italy may perhaps be ascribed."

| States. | Births. | | |
|---|---|---|---|
| | Total. | Legitimate. | Illegitimate. |
| Sardinia - - | 1,457,493 | 1,427,019 | 30,474 |
| Sweden - - | 476,799 | 445,510 | 31,289 |
| Norway - - | 181,363 | 169,252 | 12,111 |
| England - - | 517,739 | 482,943 | 34,796 |
| Belgium - - | 138,135 | 128,781 | 9,354 |
| France - - - | 982,896 | 912,968 | 69,928 |
| Prussia - - - | 591,505 | 549,376 | 42,129 |
| Denmark - - | 64,376 | 58,356 | 6,020 |
| Hanover - - | 55,559 | 50,072 | 5,487 |
| Austria - - | 894,711 | 792,890 | 101,821 |
| Wurtemburg - - | 75,456 | 66,597 | 8,859 |
| Saxony - - | 70,094 | 59,582 | 10,512 |
| Bavaria - - | 149,185 | 118,456 | 30,729 |

The mortality among illegitimate children is, in many countries, 60 or 70 per cent. higher than among those born in wedlock.* This excess is due, in part at least, to the existence of Foundling hospitals—which serve to attract, receive, and (as statistical facts demonstrate) *destroy* the hapless young creatures admitted into them.

"If the mortality were not greater among them than among legitimate children, every fifteenth person in England must be of illegitimate extraction. But the mortality is, as in other countries, no doubt greatly above the average; for without any crime whatever of his own, the illegitimate child is often exposed to dangers, hardships, and ignominy from his infancy; the law pronounces him *filius nullius*; he, however, escapes in England the tender mercies of the Foreign Foundling Hospital, and in our great towns and colonies has probably a better chance of attaining the station to which his personal conduct may entitle him than in any other country in Europe."

The Registrar-General quotes the following judicious observations from the *Handbuch der Populationistik* of Bernoulli, one of the ablest statistical writers of the present day. "The proportion of illegitimate children cannot serve as a standard of morality; nevertheless a remarkable frequency of such children is without doubt in many respects a great evil. The invariable fact that the mortality among the illegitimate is far greater than among the legitimate, and that many more of them are still-born, shows clearly enough how much more unfavourable their position is from the first. Who can doubt that their bringing up is much harder and more difficult? that the existence of a class of men, bound to society by few or no family ties, is not a matter of indifference to the State? The great majority of foundlings are illegitimate, which of itself shows how little, as a general rule, the mothers can or will care for these children. It is beyond doubt that fewer illegitimate children grow up to

* A larger proportion of the former are also still-born.

maturity; that they get through the world with more trouble; that more of them are poor; and that, therefore, more of them become criminals. Illegitimacy is in itself an evil to a man; and the State should seek to diminish the number of these births, and carefully inquire to what circumstances any increase is to be ascribed."

*Deaths.*—The average annual mortality of the population of England in the five years, 1838-42, was 2·209 per cent. or nearly *one* in every 45 individuals: in 1842, it was nearly *one* in 46. On comparing the rate of mortality in England with the mortality of France, Prussia, Austria, and Russia, it appears to be lower in this country than in either of the four States; but it must be remembered that Scotland and Ireland are not included, no steps having hitherto been taken for registering and abstracting the Births and Deaths in those parts of the United Kingdom.

*Deaths Registered in the whole, or in parts of five States of Europe.*

| | 1838 | 1839 | 1840 | 1841 | 1842 | |
|---|---|---|---|---|---|---|
| England - - - | 342,547 | 338,979 | 359,634 | 343,847 | 349,519 | Still-born not Registered. |
| France - - - | 846,199 | 780,600 | 816,486 | 804,762 | 836,152 | |
| Austria (except Hungary, &c.) | .. | 639,737 | 649,410 | 633,600 | 682,208 | Still-born Subtracted. |
| Prussia - - - | 371,990* | 408,411 | 396,494 | 392,502 | .. | Ditto. |
| Russia (part of) - | .. | .. | .. | .. | 1,856,183 | |

* Nearly.

| | Year. | Population. | Annual Deaths. | | Annual Mortality. | |
|---|---|---|---|---|---|---|
| | | | Years. | Numbers. | Per Cent. | Living to 1 Death. |
| England - - - | 1841 | 15,927,867 | 1838-42 | 346,205 | 2·207 | 45 |
| France - - - | 1841 | 34,213,929 | 1838-42 | 816,840 | 2·397 | 42 |
| Prussia - - - | 1840 | 14,928,501 | 1838-41 | 392,349 | 2·658 | 38 |
| Austria - - - | 1840 | 21,571,594 | 1839-42 | 651,239 | 2·295 | 33 |
| Russia - - - | 1842 | 49,525,420 | 1842 | 1,856,183 | 3·590* | 28 |

* Corrected for still-born *supposed* to be included in the 1,856,183 deaths; the still-born have been subtracted from the deaths in Austria and Prussia; they are probably not included in the deaths of France, and are not registered in England.

From the introductory report of the Register-General, we pass on to the letter of Mr. Farr on the state of the Public Health in 1842, &c. &c. The mortality in that year was nearly the same as in the preceding one. The mortality by diseases of the Zymotic* class—in which are comprehended all epidemic, endemic, and contagious diseases—was 4062 in 1842, and 4049 in 1841, to a million living. In 1840 it was 4947.

" The mortality in 1842 was nearly the same as in the previous year. The mortality by diseases of the Zymotic class was 4062 in 1842, and 4049 in 1841 to a million living. In 1840 it was 4947.

" *Small-pox* and *Scarlatina* present the greatest variations in the five years over which the Abstracts extend : for 16,268 persons died of small-pox in 1838, and 2715 in 1842. The epidemic of scarlatina attained its maximum in 1840, and afterwards declined.

| Total Deaths registered in | 1838 | 1839 | 1840 | 1841 | 1842 |
|---|---|---|---|---|---|
| From Small-pox . . . | 16,268 | 9,131 | 10,434 | 6,368 | 2,715 |
| From Scarlatina . . . | 5,802 | 10,325 | 19,816 | 14,161 | 12,807 |
| | Deaths to 1,000,000 living. | | | | |
| From Small-pox . . . | 1,101 | 604 | 679 | 408 | 172 |
| From Scarlatina . . . | 393 | 683 | 1,289 | 908 | 809 |

" The reduction in the mortality from small-pox since 1840 was probably the result, at least in part, of the Vaccination Act; which has not, however, yet effected all the good that may be anticipated, as an epidemic has since 1842 broken out in the metropolis, and proved fatal to great numbers. The deaths in France from small-pox were 3317 in 1842, or not more than 91 to a population of 1,000,000. The deaths in Austria from small-pox were 4619 in 1840, 5189 in 1841, and 4411 in 1842, out of a population of 21,571,594; while the deaths in England, where vaccination was discovered by Jenner, were 10,434 from small-pox in 1840, and 16,268 in 1838, out of a population under 16 millions! *Diarrhœa, Dysentery,* and *Cholera* were fatal to 7622 persons, which is considerably above the average, and the excess may be ascribed, perhaps, to the higher temperature of the year. *Influenza,* on the contrary, and probably for the same reason, was less prevalent than in the previous year; 1659 deaths were referred to that head in 1841, and 883 in 1842. *Fifteen* deaths from *Hydrophobia* occurred in 1842, while 7 only were registered in 1841; but 24 persons died of hydrophobia in 1838.

The following tables will enable the reader to determine at a glance the actual and relative amounts of mortality from different diseases, in five consecutive years.

---

* This appellation is nearly equivalent to the English words *fermented* or *fermentable,* and indicates at once the pathological view taken of their nature. It is derived from ζυμοω, I *ferment* :—this, as well as ζυμωσις, *fermentation,* occur in the writings of Hippocrates. For further particulars on this subject, and on the ingenious speculations of Mr. Farr on the nosological classification of diseases, see the Medico-Chirurgical Review for October, 1843.

| CAUSES OF DEATH. | DEATHS. | | | | | Annual Number of Deaths to 1,000,000 living. | | | | |
|---|---|---|---|---|---|---|---|---|---|---|
| | 1838 | 1839 | 1840 | 1841 | 1842 | 1838 | 1839 | 1840 | 1841 | 1842 |
| All Causes...................... | 342,529 | 338,979 | 359,561 | 343,847 | 349,519 | 22,380 | 21,856 | 22,878 | 21,589 | 21,654 |
| Specified Causes .................. | 330,559 | 330,497 | 351,757 | 336,664 | 342,774 | .. | .. | .. | .. | .. |
| I. Zymotic Diseases ........... | 67,877 | 65,343 | 76,064 | 63,148 | 64,295 | 4,596 | 4,321 | 4,947 | 4,049 | 4,062 |
| Sporadic Diseases:— | | | | | | | | | | |
| II. Of Uncertain or Variable Seat.. | 44,232 | 46,362 | 48,396 | 48,053 | 49,216 | 2,995 | 3,066 | 3,148 | 3,082 | 3,109 |
| III. Of the Nervous System ...... | 49,704 | 49,215 | 50,768 | 49,593 | 50,625 | 3,365 | 3,255 | 3,302 | 3,180 | 3,198 |
| IV. Of the Respiratory Organs .... | 90,823 | 90,565 | 92,907 | 92,183 | 92,994 | 6,149 | 5,989 | 6,043 | 5,911 | 5,875 |
| V. Of the Organs of Circulation .. | 3,562 | 3,788 | 4,370 | 4,546 | 4,925 | 241 | 250 | 284 | 292 | 311 |
| VI. Of the Digestive Organs ...... | 19,306 | 20,767 | 22,525 | 22,398 | 23,587 | 1,307 | 1,373 | 1,465 | 1,436 | 1 ,490 |
| VII. Of the Urinary Organs........ | 1,651 | 1,534 | 1,697 | 1,650 | 1,866 | 112 | 101 | 110 | 106 | 118 |
| VIII. Of the Organs of Generation .. | 3,263 | 3,412 | 3,623 | 3,555 | 3,340 | 221 | 226 | 236 | 228 | 211 |
| IX. Of the Organs of Locomotion.. | 2,102 | 2,020 | 2,167 | 2,289 | 2,272 | 142 | 134 | 141 | 147 | 144 |
| X. Of the Integumentary System.. | 420 | 448 | 525 | 528 | 497 | 28 | 30 | 34 | 34 | 31 |
| XI. Old Age..................... | 35,564 | 35,063 | 36,793 | 37,253 | 37,819 | 2,408 | 2,319 | 2,393 | 2,389 | 2,389 |
| XII. External Causes;—Poisoning, Asphyxia, Injuries ........ | 12,055 | 11 ,980 | 11,922 | 11,468 | 11,338 | 816 | 792 | 775 | 735 | 716 |

In the first, or Zymotic class, are comprehended Small-pox, Measles, Scarlatina, Hooping-cough, Croup, Thrush, Diarrhœa, Dysentery, Cholera, Influenza, Ague, Remittent fever, Typhus, Erysipelas, Syphilis, and Hydrophobia. This grouping together is surely very arbitrary, and more discordant from the ordinarily-adopted arrangements than need be. Is Croup, for example, more of a *zymotic* disease than Bronchitis (that is put in the 4th class), Thrush than Quinsey (in the 4th also), Erysipelas than Purpura (in the 2nd), or Diarrhœa than Gastritis (in the 6th)?

The second class also—designated as the group of Sporadic diseases, "of uncertain or variable seat"—contains a somewhat anomalous commixture of maladies. These are inflammation, Hæmorrhage, Dropsy, Abscess, Mortification, Purpura, Scrofula, Carcinoma, Tumour, Gout, Atrophy, Debility, Malformations, and Sudden Deaths. The only remark that we shall make on this arrangement is, that Gout might surely have been put into the 9th class, along with Arthritis and Rheumatism, rather than grouped with Purpura and Malformations. Might not Dropsy too be more appropriately regarded as a disease of the organs of Circulation than as a malady "of uncertain and variable seat?" But, indeed, it may be truly said that whatever nosological arrangement be adopted, it must necessarily be imperfect and open to objections.

| Causes of Death. | Deaths. | | | | |
|---|---|---|---|---|---|
| | 1838. | 1839. | 1840. | 1841. | 1842. |
| 1 Consumption | 59,025 | 59,559 | 59,923 | 59,592 | 59,291 |
| 2 Convulsions | 26,047 | 25,408 | 25,770 | 24,563 | 25,488 |
| 3 Pneumonia | 17,999 | 18,151 | 18,582 | 17,997 | 19,036 |
| 4 Typhus | 18,775 | 15,666 | 17,177 | 14,846 | 16,201 |
| 5 Debility | 12,634 | 15,143 | 16,225 | 16,189 | 17,339 |
| 6 Scarlatina | 5,802 | 10,325 | 19,816 | 14,161 | 12,807 |
| 7 Dropsy | 12,342 | 12,251 | 13,261 | 13,095 | 12,724 |
| 8 Small pox | 16,268 | 9,131 | 10,434 | 6,368 | 2,715 |
| 9 Violent deaths | 11,727 | 11,632 | 11,594 | 11,100 | 11,092 |
| 10 Measles | 6,514 | 10,937 | 9,326 | 6,894 | 8,742 |
| 11 Hooping-cough | 9,107 | 8,165 | 6,132 | 8,099 | 8,091 |
| 12 Hydrocephalus | 7,672 | 7,749 | 8,000 | 7,973 | 8,057 |
| 13 Gastritis and Enteritis | 6,061 | 6,524 | 7,260 | 6,980 | 7,249 |
| 14 Apoplexy | 5,630 | 5,293 | 5,451 | 5,581 | 5,362 |
| 15 Teething | 4,404 | 5,016 | 5,219 | 5,324 | 5,689 |
| 16 Paralysis | 4,975 | 4,910 | 5,490 | 5,495 | 5,559 |
| 17 Croup | 4,463 | 4,192 | 4,336 | 4,177 | 4,457 |
| 18 Inflammation | 5,816 | 4,940 | 3,965 | 3,306 | 3,178 |
| 19 Heart, disease of | 3,319 | 3,551 | 4,058 | 4,246 | 4,661 |
| 20 Sudden death | 3,012 | 3,696 | 3,610 | 3,901 | 3,802 |
| 21 Diarrhœa | 2,482 | 2,562 | 3,469 | 3,240 | 5,241 |
| 22 Atrophy | 2,018 | 2,142 | 3,013 | 3,535 | 3,970 |
| 23 Childbirth | 2,811 | 2,915 | 2,989 | 3,007 | 2,687 |
| 24 Lungs, disease of | 2,568 | 2,551 | 2,737 | 2,788 | 2,944 |
| 25 Liver, disease of | 2,590 | 2,762 | 2,681 | 2,706 | 2,704 |
| 26 Carcinoma | 2,448 | 2,691 | 2,786 | 2,746 | 2,941 |
| 27 Cephalitis | 2,178 | 2,368 | 2,581 | 2,498 | 2,456 |
| 28 Hydrothorax | 2,306 | 2,149 | 2,345 | 2,282 | 2,127 |
| 29 Bronchitis | 2,067 | 1,663 | 2,053 | 2,267 | 2,627 |

In this Table—which we have formed out of the lengthened and detailed one (No 1) given by Mr. Farr—the diseases have been arranged according to their relative fatality. We have limited it, it will be seen, to such diseases only as occasion a mortality of, and above, 2000 per annum: all others being excluded.

A few cursory remarks, in the way of comment, may be appropriately introduced here. The average mortality from Bronchitis—one of the most frequent and frequently fatal of diseases—is not much above 2,000 annually; whereas that from Asthma is set down at between 5 and 6,000, and that from Gastritis and Enteritis at between 6 and 7,000. This is certainly not what we should have expected; but then it is to be noticed that a large proportion of the fatal cases of Pneumonia—no fewer than about 18,000 per annum—might probably have been referred with greater propriety to Bronchitis. Again, is it not by inflammation of the air-tubes that Pertussis generally proves fatal?

The loss from Influenza (about 1,000 per ann.) is surprisingly small; but then that from Debility is immense.

Among the diseases "of uncertain or variable seat," there are no fewer than about 4000 deaths per annum attributed to that most vague of nosological terms, *inflammation*. Inflammation of what?—the reader will naturally ask. Not of the skin; for Erysipelas has a place in the first groupe. Not of the viscera; for each viscus has its own inflammatory member. Not of the joints; for Gout, Arthritis, and Rheumatism will naturally claim that as their own.

The mortality, set down to the account of dropsical disease, is immense. First of all we have Dropsy, the general term; then Hydrocephalus, Hydrothorax, Ascites, and Ovarian Dropsy;—these various forms of one disease causing death to the amount of nearly 23,000, in the course of the year.

The annual number of deaths from Gout does not exceed 200; but then it is to be remembered that not a few of the sudden deaths, especially in old people, are attributable to gouty affections of the heart and stomach. There is an 'Arthritis' set down as distinct from Gout on the one hand, and from Rheumatism on the other; we do not quite understand what to make of it. The mortality from this disease does not exceed between 30 and 40 per annum.

That from Rheumatism is nearly 1,000; and yet we are told—and properly so—in medical books, that this disease is very rarely fatal: its effects or consequences—of which cardiac lesions are perhaps the most frequent—are often so, but not the primary disease itself.

The mortality from Convulsions—we suppose, in infancy and early youth chiefly—is very high; that from Epilepsy, which stands by itself, is only between 1,000 and 1100. From 25 to 30 deaths per annum are attributed to Chorea: if this be correct, how comes it that we know so little—next to nothing—of the pathology of this disease?

In the Respiratory groupe, after Consumption and Pneumonia, Asthma stands next on the list, in point of fatality. A vast number of the deaths however from this disease are unquestionably attributable to some organic diseases of the Heart or Lungs.

The mortality from Peritonitis is set down so low as between 2 and 300 a year. Puerperal fever does not occur in the catalogue at all; and we had therefore supposed that it might have been comprehended under the generic term of Typhus; but we afterwards found out that it was classed under the head of Child-birth—the deaths from which cause nevertheless fall short of 3000 per annum.

While Peritonitis is so very sparingly fatal, we were surprised to learn that nearly 700 deaths annually are attributed to Worms. Surely there must have been Remittent Fever, Tabes Mesenterica, or Atrophy at the same time, to produce so large a mortality. One remark more, and we close.

What a marked contrast does British present to Indian Pathology! For, whereas Diseases of the Liver constitute nearly a half of the mortality in Bengal, the number of deaths, in the table before us, from Hepatitis, Jaundice and Disease of the Liver does not exceed, in all, 4000 out of an annual mortality of between 3 and 400,000, in England.

We had concluded the preceding notice of the Registrar-General's Report, when we received the very valuable one by Mr. Martin, on the condition, in reference to hygiene, of certain large towns in England, which he had visited officially as one of the members of the "Health of Towns' Commission." We trust, for the benefit of Parliament and of the public generally, that the other Commissioners may perform their duties with as much zeal and ability as Mr. M. has done. He has evidently felt the importance of his task, and, by applying the energies of his vigorous and enlightened mind to its execution, has produced an admirable report on the sanatory condition of (part of) the working-classes of our population. We have extracted some of its most interesting statements, recommending strongly the general remarks, with which it closes, to the attentive perusal of all who wish to make themselves acquainted with the important subject of Medical Police.

The difference in the amount of mortality in the agricultural, compared with that in the manufacturing districts and towns in England is most striking. The ratio ranges from one death in 54 to one death in 29 of the inhabitants annually;—a deplorable example and proof of the difference in the physical condition and comfort of the respective classes of our labouring population. It is reckoned moreover that, out of every 1000 births, 221 only die under five years of age, in our agricultural districts; while not fewer than 385 die annually, under the same period of life, in all of our closely-built towns. This is a sad reflection; and yet no one can doubt for a moment but that the amount of mortality among our artisans might be most sensibly diminished by the introduction of appropriate sanatory regulations. When we remember that in Birmingham there are at least 33,000, in Manchester 83,000, and in Liverpool no fewer than 100,000, human beings compressed within the compass of a single square mile, and a vast proportion too of these inhabitants living in squalor, wretchedness and vice, how can we be surprised at the high rate of mortality in such places, more especially in the last-named town, our great western sea-port? Read what Mr. Martin says on this subject:

"Resuming the comparison again, we find that, in a thousand deaths in the country districts, 202 persons the age of 70 years; while in Liverpool, for instance, but 90 persons out of 1000 attain to the same age; and while the average age at death in agricultural Rutlandshire is 38 years, it is stated to be but 21 years in Liverpool. Taking the same population, it has been shown by the Registrar-General that in four years a greater number died in town districts than in country districts by 99,752. Again, out of 1,000,000 of persons living, there occurs annually in the country, and where the population to the square mile is but 199 persons, 19,300 deaths; but in towns and where the population to the square mile is 5108 persons, there occur 27,073 deaths. We find also that fever —the great disease of adolescence and manhood—the disease that most afflicts men and women at the most useful and valuable period of life—the great destroyer of mankind in every climate—is bred and propagated in an especial manner in large towns; that towns present exactly in proportion as they are closely built and inhabited, the largest proportion of sickness and death from fever, not only as compared with the population, but with the total number of deaths from all causes. The fevers of the crowded quarters of London and of all the great towns is annually assuming a more formidable character, with an increase of its contagious virulence and power of propagating itself; its type everywhere indicating increased depression in the powers of life, as shown by the progressive lowering in the tone of the nervous and vascular systems."

Besides the continually devastating effects of Fever among the lower classes of the population in large towns, we find that almost every disease, without exception, is more fatal in the manufacturing than in the agricultural districts of our land. So much is this the case, that the increase of deaths among children is four-fold by epidemics, and nearly ten-fold by convulsions, in towns as compared with the country. Among adults too, the prevalent epidemic diseases are more than thrice as fatal in Liverpool and Manchester than in the country; while the deaths by diseases of the lungs are nearly doubled, those by diseases of the nervous system are as 5½ to 1, and by diseases of the digestive organs as 2½ to 1. All observation, continues our intelligent author, goes to demonstrate that the liability to Consumption increases in an enormous ratio with the increase of crowding and its accompaniment, defective ventilation.

Mr. Martin shews most convincingly, by a reference to the present ratio of mortality in the British navy (about 1·6 per cent. per annum) compared with what it was 40 or 50 years ago (7·2 per cent.), how much may be done, for the improvement of the public health, by judicious administrative measures; and he avails himself of the opportunity of paying a well-merited compliment to the labours of the medical officers of our public services, in this most important field of professional enquiry. "It is not," he remarks, "too much to say that the merit of taking decisive measures for the abatement of this and other injurious influences, is due to the surgeons of our fleets and armies, wherein are now to be found the best and most extended examples of the benefits to be derived from a regulated and supervised plan of sanatory regulation applied to masses. The value of the knowledge, derived from the experience of the medical officers of the public services, has been practically though silently felt in every civil community, whether in Europe or America; but the merits of the authors have hardly in any instance, or at any time, been recognized in this country. This becomes the more obvious when the fact is borne in mind that, up to this day, there is not a principle in preventive medicine that has not been long since indicated in the writings of Pringle, Lind, Blane, and of Drs. Robert Jackson, William Fergusson, and James Johnson."

That certain trades and occupations are injurious to the health of artisans is not to be disputed; but the grand cause of the fearful ravages of disease in our manufacturing districts is unquestionably to be traced to the filth and wretchedness of their dwellings, the insufficient supply or the bad quality of their food, their want of cleanliness arising from a defective supply of water, and the vicious habits that so generally—we had almost said necessarily—accompany personal and domestic discomfort. Some of the foreign governments have set an excellent example to ours in this respect, by the active sanatory regulations which they have established among their manufacturing and labouring classes; and amply have they been repaid for the performance of this most important duty, by the improvement in the state of the general health of their artisans. The cotton factories near Vienna are alluded to, as affording a striking illustration of the truth of this remark. Even in a mere financial point of view, it would be well if there was greater attention paid to the state of the dwellings and the general habits of our working classes. It is generally easier, and therefore cheaper in the long run, to ward off an evil than to remedy it, to prevent a disease than to cure it. If this be true in reference to private families, how much more is it in the case of large masses of men, crowded together in wretchedness and filth, without means of obtaining proper relief, and—a consideration of the highest hygienic importance—each one of whom often becomes a new source of morbific diffusion. The following remarks, on "the various losses occasioned to the public finances by *preventible diseases*," are full of force and truth:

"It has been calculated that the total number of orphan children, on account of whose destitution relief was given from the poor-rates in the year ending Lady-day, 1840, was 112,000. Of the parents of this number, we are confident that accurate investigation would demonstrate full one-half died of preventible disease.—The loss to the industrious classes consequent on sickness alone, has been variously estimated. One of the lowest calculations, rates the number of days of sickness in the year, experienced by a man, his wife, and two children above 12 years of age, at 29 days, or about one-thirteenth of the entire year. Estimating the weekly earnings of such a family at 40*s*., we have here a great loss by labour alone, without medicine and other contingent expenses.—'But this is vastly below the mark, although quite enough to prove how truly economical it would be in every way, to expend the same money upon airy, salubrious lodgings, conducive at once to health, morals, and respectability. In fact, there can be no doubt that the enormous sums spent every year in hospitals, infirmaries, and union workhouses, are incomparably greater than the expenditure necessary for preventing disease and pauperism.' This I believe to be true and easy of proof: indeed, we have only to turn to the singularly valuable 'Report' of Mr. Chadwick, to perceive as clearly as need be, how vast are the charges on account of sickness and mortality which are of easy prevention—how enormous the charges on the reduced duration of life—on the reduction by sickness of the periods of working ability or production—on the machineries for the suppression of much of the vice and crime, which comes within the province of the police—as well as for the relief of much of the destitution which comes within the province of the administrator's relief. According to the rate ascertained in eight Unions, Mr. Chadwick concludes that, in all the Unions, about 27,000 cases of premature widowhood occur, and more than 100,000 cases of orphanage—all which may be ascribed to removeable causes. Mr. Hawksley estimates the loss in Nottingham alone, 'by the pressure of removeable causes of sickness and mortality at 300,000*l*. per annum.' It is quite unnecessary to pursue this subject farther. Innumerable details in proof are now before the public, and further description would but weaken the effect."

Mr. Martin, after shewing how much might be done by a system of more stringent parliamentary enactments and police regulations respecting the removal of all refuse and filth, the thorough ventilation of lanes and dwellings, the abundant supply of water for the purposes of washing as well as of food, the improvement of the sewerage, the removal of burial-places to the environs, the establishment of public baths, and of parks and open play-grounds, &c. concludes his Report with some admirable observations on the reciprocal influence of morals on public health, and of destitution and defective diet on the moral character. We give a sample:

"I have said nothing directly on the influence of morals on health—a large and pervading subject—on which, however, it is proper to touch, because it is proved by every day's experience, that, in the large majority of instances, immorality and its consequent diseases are but the results of physical destitution or depression. How commonly do we find that the deep and protracted distress of mind which necessarily accompanies aggravated states of bodily suffering, produces that diseased action and reaction—that reciprocal moral and physical disturbance—which, sooner or later, destroys the balance of health. It is this double action that is tearing up the nervous system of the operatives—that system which, from its highly endowed sensibility, receives the first impress of every morbific cause, whether acting primarily on the mind or the body. The more impressible character of the nervous system in females, rendered less apparent by the passive fortitude of their nature, and by their greater power of endurance, comparatively, brings upon mothers, and through them upon their offspring, a mass of suffering greater even than that which afflicts the more complainant father.

"I am here stating important circumstances that have come largely under my observation. I have no desire to excite useless sympathy. It is proved that, by lowering the power of the nervous system, the mind is broken down even more rapidly than the body. It is proved that, so long as the physical powers are depraved and depressed, there can be no hope of moral improvement; and these facts, open to the observation of all, ought to be sufficient."

They ought indeed to be sufficient; and most fervently do we trust that the Government of this country will immediately enter upon the beneficent work of legislating for the hygienic condition of the poor, and may ere long have washed off the reproach—too justly made against us as a nation—of being more ready to punish crime than to reform the criminal, to deter by penal enactments than to encourage by the mild influences of paternal authority.

